# Three-Dimensional Algebraic Models of the tRNA Code and 12 Graphs for Representing the Amino Acids

**DOI:** 10.3390/life4030341

**Published:** 2014-08-11

**Authors:** Marco V. José, Eberto R. Morgado, Romeu Cardoso Guimarães, Gabriel S. Zamudio, Sávio Torres de Farías, Juan R. Bobadilla, Daniela Sosa

**Affiliations:** 1 Theoretical Biology Group, Instituto de Investigaciones Biomédicas, Universidad Nacional Autónoma de México, México D.F. 04510, Mexico; E-Mails: gazaso92@gmail.com (G.S.Z.); jromanb@unam.mx (J.R.B.); dani.dansp16@gmail.com (D.S.); 2 Facultad de Matemática, Física y Computación, Universidad Central “Marta Abreu” de Las Villas, Santa Clara, Cuba; E-Mail: morgado@uclv.edu.cu; 3 Laboratório de Biodiversidade e Evolução Molecular, Departamento de Biologia Geral, Instituto de Ciências Biológicas, Universidade Federal de Minas Gerais, 31270.901 Belo Horizonte, MG, Brazil; E-Mail: romeucardosoguimaraes@gmail.com; 4 Centro de Ciencias Exactas y Naturales, Universidade Federal da Paraíba, João Pessoa, Brazil; E-Mail: stfarias@yahoo.com.br

**Keywords:** standard tRNA code, human tRNA code, graphs of amino acids, algebraic models, symmetry, evolution genetic code, polar requirement

## Abstract

Three-dimensional algebraic models, also called Genetic Hotels, are developed to represent the Standard Genetic Code, the Standard tRNA Code (S-tRNA-C), and the Human tRNA code (H-tRNA-C). New algebraic concepts are introduced to be able to describe these models, to wit, the generalization of the 2^n^-Klein Group and the concept of a subgroup coset with a tail. We found that the H-tRNA-C displayed broken symmetries in regard to the S-tRNA-C, which is highly symmetric. We also show that there are only 12 ways to represent each of the corresponding phenotypic graphs of amino acids. The averages of statistical centrality measures of the 12 graphs for each of the three codes are carried out and they are statistically compared. The phenotypic graphs of the S-tRNA-C display a common triangular prism of amino acids in 10 out of the 12 graphs, whilst the corresponding graphs for the H-tRNA-C display only two triangular prisms. The graphs exhibit disjoint clusters of amino acids when their polar requirement values are used. We contend that the S-tRNA-C is in a frozen-like state, whereas the H-tRNA-C may be in an evolving state.

## 1. Introduction

The transfer RNA (tRNA) is perhaps the most important molecule in the origin and evolution of the genetic code. Just two years after the discovery of the double helix structure of DNA, F. Crick [1,2] proposed the existence of small adaptor RNA molecules that would act as decoders carrying their own amino acids and interacting with the messenger RNA (mRNA) template in a position for polymerization to take place. A small nucleic acid (perhaps RNA) could serve the role of an adaptor, one part of the adaptor molecule binding a specific amino acid and another part recognizing the nucleotide sequence encoding that amino acid in an mRNA [3,4]. These adaptors, larger than predicted by Crick, are the tRNAs soon discovered in 1958 [5]. The tRNA adaptor “translates” the nucleotide sequence of an mRNA into the amino acid sequence of a polypeptide. The overall process of mRNA-guided protein synthesis is often referred to simply as translation. The coding properties of each tRNA are not determined by the amino acid it carries but by the interaction of the aminoacylated tRNA with the mRNA template. The set of interactions between aminoacyl-tRNA synthetases (aaRSs) and tRNAs is considered to be the Second Genetic Code [6]. This operational code comprises the set of signals or rules by which aaRSs recognize their cognate tRNAs [6,7,8]. The first genetic code to appear during evolution was not the Standard Genetic Code (SGC), but rather this second genetic code. The two domains of the L-shaped tRNA would have arisen independently, with the acceptor branch appearing first. In a later stage in history, the catalytic cores of synthetases emerged independently in their class I and II versions. Co-evolution of catalytic cores of synthetases and accepting hairpins led to an operational RNA code that associated specific amino acids with hairpin structures [9]. The anticodon domain of tRNA and the additional domains of synthetases appeared later in evolution. Anticodon domains brought the link between the RNA operational code and the correlated tRNA recognition by synthetases with the anticodon-dependent recognition by mRNA. Proposals on a wider role of tRNAs in the origins of the whole system of template-directed biopolymers were implemented by Eigen and Winkler-Oswatitsch [10], soon followed by Bloch *et al.* [11].

The SGC has been theoretically derived from a primeval RNY genetic code under a model of sequential symmetry breakings [12,13,14], and vestiges of this primeval RNY genetic code were found in current genomes of both Eubacteria and Archaea [15]. All distance series of codons showed critical scale invariance not only in RNY sequences (all ORFs concatenated discarding the non-RNY triplets), but also in all codons of two intermediate steps of the genetic code and in all kind of codons in the current genomes [15]. Such scale invariance has been preserved for at least 3.5 billion years, beginning with an RNY genetic code to the SGC throughout two evolutionary pathways. These two likely evolutionary paths of the genetic code were also analyzed algebraically and can be clearly visualized in three, four and six dimensions [13,14].

In this work, we focus on the codon-anticodon rules for which we develop algebraic models. In previous works [12,13], algebraic models were rigorously derived in order to represent the SGC. The three-dimensional (3D) models were dubbed Genetic Hotels of codons and Hotels of amino acids [14]. These models were used to test hypotheses about the evolution of the SGC [15]. In the present work, we compare the SGC with both the Standard tRNA code (S-tRNA-C) and the Human tRNA code (H-tRNA-C). The tRNA code that is considered to be Standard comprises 46 tRNAs, due to the absence of triplets with the base A in the 5’ position and of the tRNAs corresponding to the stop codons; the initiator tRNA is added to the elongation set of 45 [16]. Wobbling anticodons recognize more than one codon. Wobble reduces the number of required anticodons substantially. In the standard set of tRNAs the anticodon triplets that begin with A either have their genes excluded from the genomes or, when there are genes for them, the A is modified into a functional base and the genes coding for tRNAs with anticodons beginning with G are excluded. The net result is that anticodons beginning with purines (R) are always of only one kind for one amino acid; this is usually G, sometimes a modified purine, rarely A [17]. This salient wobbling feature allows two or more neighboring codon triplets to share a common anticodon, which is the reverse complementary of one of them. In the human tRNA set [18], there are 16 triplets whose anticodons are not the reverse complementary of the corresponding codons, but the reverse complementary of a neighboring triplet, which differs from it, only in the third nucleotide. In other words, in the H-tRNA-C there are 16 vacant triplet sequences in the 5’R sets: eight of the remaining begin with A, which is the base transcribed from the genes, but is later modified into Hypoxanthine that is part of the nucleoside Inosine (I), while the other eight begin with G [19]. In this work, we elaborate 3D algebraic models, *i.e.*, Hotels of the Anticodons of tRNA for both the S-tRNA-C and the H-tRNA-C. In both the Standard and Human Hotel of Anticodons, the 45–46 anticodons are distributed in 18 subcubes or condominiums in 3D.

The manuscript is organized as follows. First, we highlight some fundamental algebraic properties of the SGC in 3D. Second, we review some fundamental biological properties of the tRNA code for both the S-tRNA-C and the H-tRNA-C. Third, we develop three-dimensional algebraic models for both codes. New algebraic concepts are introduced to be able to describe these models, to wit, the generalization of the 2^n^-Klein Group and the concept of a subgroup with a tail. Next we demonstrate that there are exactly 12 ways to represent a graph of amino acids depending on the ordering of the four RNA nucleotides. Twelve phenotypic graphs of amino acids are calculated for each genetic code. The centrality measures of each of the 12 graphs for each genetic code are calculated and their averages are statistically compared. A common subgraph of connected amino acids that corresponds to a triangular prism is encountered in the SGC, the S-tRNA-C, and in the H-tRNA-C. We also searched for matches between the topology of the networks with physicochemical properties of amino acids. Finally, the present results are discussed in the context of algebraic models of the evolution of the genetic code.

## 2. Standard Genetic Code

Group theory is the branch of mathematics that is used for studying symmetry. In mathematics, a group is an algebraic structure consisting of a set together with an operation that combines any two of its elements to form a third element.

### 2.1. The Concept of a Group

**Definition 1:** A group is an algebraic system consisting in a nonvoid set G, with a binary operation × where the followings axioms hold:

(1) Associativity: x × (y × z) = (x × y) × z for all x, y, z, elements of G.
(2) Existence of neutral: There is an element e in G such that, x × e = e × x = x for all x.
(3) Existence of inverses: For every x of G there is an element x^−^^1^ called the inverse of x, such that x × x^−^^1^ = x^−^^1^ × x = e.

In physics, groups are important because they describe the symmetries, which the laws of physics seem to obey. According to Noether’s theorem, every symmetry of a physical system corresponds to a conservation law of the system. Analogously, a large part of our work has contributed to determine the symmetry groups that not only describe the structure of the SGC but also its evolution [12,13,15]. The genetic code seems to have evolved from a primeval code by means of symmetry breakings [12,15,20]. The genetic code can be broken down into a product of simpler groups reflecting the pattern of degeneracy observed and the salient fact that evolution did not erase its own evolutionary footsteps. A symmetrical primeval genetic code, specifying eight different amino acids, and two symmetrical intermediate codes, between the primeval and the SGC, specifying each 15 different amino acids have been found [12,15]. The geometrical and algebraic representations of the primeval RNA code, intermediate RNA codes, as well as the SGC in three (Genetic and Phenotypic Hotels), four, and six dimensions have facilitated to test several hypothesis of the origin and evolution of the SGC [12,13].

In the next sections we derive the symmetry groups of the Genetic Hotels of the SGC, the S-tRNA-C and the H-tRNA-C.

### 2.2. Some Basic Definitions and Notations

In a previous work [12], the standard genetic RNA-code was algebraically described as a three-dimensional GF(4)-vector space, where GF(4) denotes the Galois Field of four elements. The set N = {C, U, A, G} of the four RNA nucleotides is endowed with a structure of algebraic field, by means of the matching C↔00, U↔01, A↔10, G↔11, of N with the set (ℤ_2_)^2^ = ℤ_2_ × ℤ_2_ = {00, 01, 10, 11} of the binary duplets, with ℤ_2_ = {0, 1} the binary field of two elements, generally known as GF(2), the Galois Field of two elements. There is nothing special about this matching of the nucleotides. In fact the set N can be partitioned into two disjoint binary classes in three different ways, based on chemical criteria: strong-weak, amino-keto, and pyrimidine-purine, and similar results can be obtained [13]. The field structure of (ℤ_2_)^2^, identified with the polynomial ring ℤ_2_[*x*] is defined by means of the irreducible and primitive polynomial x^2^ + x + 1. The module 2 bitwise addition in the additive group (ℤ_2_)^2^ induces in the set N a group structure whose Cayley Table is (Table 1):

**Table 1:** The Four Klein Group.

+	C	U	A	G
**C**	**C**	**U**	**A**	**G**
**U**	**U**	**C**	**G**	**A**
**A**	**A**	**G**	**C**	**U**
**G**	**G**	**A**	**U**	**C**

Table 1 corresponds to the Four Klein Group, which is an Abelian group of order four, where every element is its own inverse. This means that each element, different from the neutral C, has order two. The Klein four group emerges naturally from the simplest model for the prebiotic evolution that has led to the SGC [13]. The Cartesian set N × N × N abbreviated as NNN is endowed with the structure of a three-dimensional GF(4)-vector space. Identifying the duplets 00, 01, 10, and 11 with their equivalent numerals zero, one, two and three in the base four numerical system, the set NNN is canonically embedded as a subset in the ordinary three-dimensional Euclidean vector space ℝ^3^, where the triplets UCC, CUC, and CCU correspond, respectively, to the unitary vectors e_1_ = (1, 0, 0), e_2_ = (0, 1, 0), and e_3_ = (0, 0, 1), of the so-called orthonormal canonical basis. The set NNN has the geometric shape of a cube, or regular hexahedron, the sides of which are of the length three. Due to its resemblance to a three-floor cubic building, we have called it the Genetic Hotel [14].

### 2.3. A Metric or Distance in the Hotel NNN

For the genetic hotel NNN, identified as the vector space (GF(4))^3^ with being GF(4) = {0, 1, 2, 3} the Galois field of four elements with the appropriate field operations, we use the so-called Manhattan or Taxi-cab distance. The Manhattan distance is consistent with the graph-theoretical concept of a distance between vertexes. The Manhattan distance between the two triplets (X_1_X_2_X_3_) and (Y_1_Y_2_Y_3_) of NNN is defined as the nonnegative integer |X_1_ − Y_1_| + |X_2_ − Y_2_| + |X_3_ − Y_3_|, where the operations are the ordinary addition and subtraction in the set ℤ of integers, and the vertical bars mean the usual absolute value of a real number. The latter definition of distance in NNN is similar to the one used for the definition of the Hamming distance in a hypercube (ℤ_2_)^n^, where the only scalars are zero and one. The Manhattan distance gives us the minimal number of edges or unitary segments in a path between two triplets. Obviously the greatest distance in NNN is equal to nine, the distance between the null triplet CCC and its complementary GGG. It is easy to prove that two triplets are adjacent if, and only if, they differ in only one component, and these different components are consecutive under the selected order {C, U, A, G}, such that the Manhattan distance between them, D((X_1_X_2_X_3_), (Y_1_Y_2_Y_3_)), is equal to one.

### 2.4. A Note about Isometric Transformations

The group (E(NNN,◦)) of all the symmetries of the GF(4)-vector space NNN

In the first place, the linear isometries are those represented by orthogonal matrices with respect to the canonical basis (e_1_ = (1, 0, 0), e_2_ = (0, 1, 0) and e_3_ = (0, 0, 1)). They are the so-called permutation matrices, obtained from the identity matrix I_3_=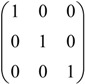 by permutations of their columns, namely, the six 3 × 3 permutation matrices: I_3_=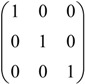, A=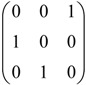, A^2^=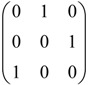, B=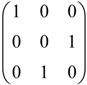, AB=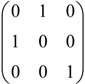, A^2^B=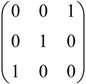.

They conform an order six group, which we will denote as P_3_(GF(4)), generated by A and B, isomorphic to the symmetric group S{3e_1_, 3e_2_, 3e_3_} of the set of the three corner triplets GCC, CGC, and CCG, that are collinear with the three unitary canonical vectors UCC = e_1_ = (1, 0, 0), CUC = e_2_ = (0, 10), CCU = e_3_ = (0, 0, 1). Among the generators A and B, the following defining relations take place: A^3^ = B^2^ = I_3_, BA = A^2^B. The group P_3_(GF(4)) is also isomorphic to the dihedral group *D*_3_ of all the symmetries of an equilateral triangle.

On the other hand, the only isometric translations are the ones associated with the eight triplets CCC, CCG, CGC, CGG, GCC, GCG, GGC, and GGG, situated at the corners of the multicube NNN. These eight translations conform an Abelian order eight group, generated by the three order two elements GCC, CGC, and CCG. We will denote this as IT(NNN) and call it the group of isometric translations of the multicube NNN. This group is isomorphic to the additive group ℤ_2_ × ℤ_2_ × ℤ_2_, with the component-wise module 2 addition. In fact, this is what we, later on, will call a generalized 2^3^-Klein Group. Hence, there are 48 isometric transformations, which are compositions of the six linear isometries with the eight isometric translations. They comprise the known 48 symmetries of a cube or regular hexahedron.

The so-called Euclidean group E(NNN) of all the isometric transformations of the multicube NNN, is the semidirect product IT(NNN)↙P_3_(GF(4)). This means that every isometric transformation of NNN may be represented, in a unique way, as a composition T_XYZ_◦M of the isometric translation T_XYZ_, where X, Y, Z ∈ {C,G} with the permutation matrix M, and IT(NNN) being a normal subgroup of E(NNN). The Euclidean group E(NNN) is generated by the five elements: A, B, T_GCC_, T_CGC_, T_CCG_, with the defining relations: A^3^ = B^2^ = I_3_, BA = A^2^B,

(T_GCC_)^2^ = (T*_C_*_GC_)^2^ = (T*_CC_*_G_)^2^ = I_3_,A◦ T_GCC_ = T_CGC_◦ A,B◦ T_GCC_ = T_GC*C*_◦ B,A◦ T*_C_*_GC_ = T_C*C*G_◦ A,B◦ T*_CG_*_C_ = T_C*CG*_◦ B,A◦ T*_CC_*_G_ = T_GC*C*_◦ A,B◦ T*_CC_*_G_ = T_*C*GC_ ◦ B, T_GCC_◦ T_CGC_ = T_CGC_◦ T_GCC_,T_GCC_ ◦ T_C*C*G_ = T_C*C*G_ ◦ T_GCC_,T*_C_*_GC_ ◦ T_C*C*G_ = T_C*C*G_ ◦ T*_C_*_GC_.

## 3. Some Remarkable Subsets of the Standard Genetic Hotel

**Figure 1:**
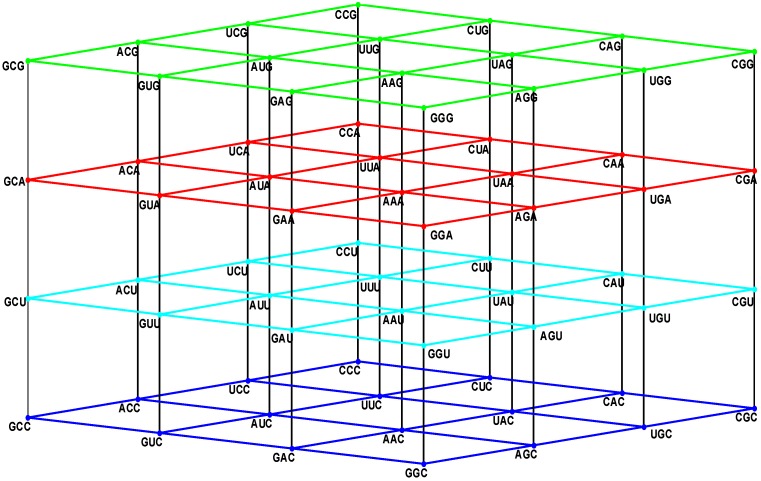
The four walls of the Standard Genetic Hotel with the Cartesian set N × N × N (NNN). Positions of the 64 codons or triplets in the Genetic Hotel. The 64 codons are distributed in eight subcubes or condominiums in three dimensions (3D). Within each condominium we can move from one triplet to its nearest neighbor by means of a transition in the first, second or third position of the codon. In order to abandon a condominium, a transversion is required. We note that there are 48 symmetries of the Hotel.

The set N is the disjoint union of two binary sets, the pyrimidines Y = {C, U} and the purines R = {A, G}. The subset YYY = Y × Y × Y is a unitary cube, which, under addition, is a subgroup of the additive group (NNN, +). It is not a GF(4)-vector subspace, since it is not closed under multiplication of scalars by vectors. It is, however, actually a ℤ_2_-vector subspace, if NNN is seen as a six-dimensional ℤ_2_-vector space, isomorphic to the binary hypercube (ℤ_2_)^6^. The multicube NNN is the union of 27 unitary cubes, which are isometric to the subcube YYY whose nucleotide components are pyrimidines. Eight out of those 27 subcubes are the group-theoretical cosets of the subgroup (YYY, +), namely, the subcubes YYY, YYR, YRY, YRR, RYY, RYR, RRY, RRR, which are the corner subcubes of the whole multicube NNN. We refer to Figure 1 in the following.

### 3.1. The Three Floors and the Roof

The multicube NNN is the disjoint union of the four sets: NNC, NNU, NNA, and NNG, which are, respectively, the first, the second, the third floor, and the roof of the hotel. The floor NNC is a two-dimensional vector subspace, generated by the unitary vectors UCC and CUC, inserted in the XY-Euclidean plane. The other three: NNU, NNA, and NNG are two-dimensional affine subspaces, or group theoretical cosets of NNC. The subset NNY, of all the triplets that end in a pyrimidine nucleotide, C or U, is the union of the first two floors: NNC and NNU. It is an order 32 subgroup of the additive group (NNN, +). Its complement, the subset NNR, of all the triplets that end in a purine nucleotide, A or G, is the union of the two floors NNA and NNG. It is the only different coset of the subgroup (NNY, +).

### 3.2. The Vertical Front Walls

The very front vertical wall is the set GNN of the triplets that begin with G. The first inner vertical wall is the set ANN of the triplets that begin with A. The second inner vertical wall is the set UNN of the triplets that begin with U. The rear vertical wall is the set CNN of the triplets that begin with C. The latter is the two-dimensional GF(4)-vector subspace, generated by the unitary vectors CUC and CCU. The other front walls, GNN, ANN, and UNN, are the affine subspaces, or group-theoretical cosets, determined, respectively, by the translations T_GCC_, T_ACC_, and T_UCC_, associated with vectors GCC, ACC, and UCC, respectively, which are orthogonal to the plane CNN, which is inserted in the YZ-Euclidean plane. The additive subgroups: (NNC, +) and (CNN, +) are isomorphic to the group (ℤ_2_)^4^ = ℤ_2_ × ℤ_2_ × ℤ_2_ × ℤ_2_, with the bitwise module 2 addition. In this latter group every element different from the neutral has order two.

Next, we will introduce a novel concept. 

### 3.3. The Concept of Generalized 2^n^-Klein Group

***Definition*:** We will call a generalized 2^n^-Klein Group to an order 2^n^ finite Abelian 2-group in which every element different from the neutral has order two. It is isomorphic to the additive group of the hypercube (ℤ_2_)^n^ with the bitwise module 2 addition. Using another genus of terminology, a 2^n^-Klein Group is a homocyclic Abelian 2-group of rank n, where every nonnull element has order two.

***Example*:** The additive group (NNN, +) of the SGC Hotel, isomorphic to the additive group of the six-dimensional hypercube (ℤ_2_)^6^, is a 2^6^-Klein Group.

From this it follows that the additive subgroups (CNN, +) and (NNC, +), associated with the rear wall CNN and the floor NNC, respectively, are generalized 2^n^-Klein Groups. Analogously, the subgroups (NNY, +) and (YNN, +) of triplets that end or begin with a pyrimidine nucleotide, respectively, are generalized 2^5^-Klein Groups.

We have observed [14] that 15 out of the 20 known primary amino acids, have representing coding triplets in the first floor NNC, while the other three, together with the stop signal, have representing coding triplets in the third floor NNA. The remainders, Trp and Met, have their only representing triplets on the roof of the building.

Note from Figure 1 that: (1) The plane NNC, blue, the first floor, is the two-dimensional vector subspace, generated by the canonical unitary vectors UCC and CUC. (2) The plane NNU, cyan, the second floor, is the two-dimensional affine subspace, determined by the vertical translation T_CCU_, associated with the unitary vector CCU. (3) The subset NNY is the union NNC ∪ NNU of the first and second floors. It consists of all the triplets that end in a pyrimidine. It is an index two subgroup of the additive group (NNN, +). (4) The plane NNA, red, the third floor, is the two-dimensional affine subspace, determined by the vertical translation T_CCA_, associated with the vector CCA of length two. (5) The plane NNG, green, the roof, is the two-dimensional affine subspace, determined by the vertical translation T_CCG_, associated with the vector CCG of length three. (6) The subset NNR is the union NNA ∪ NNG of the third floor and the roof. It consists of all the triplets that end in a purine. It is a coset of the subgroup NNY.

## 4. The tRNA Code

Today we know that an amino acid is covalently bound at the 3’ end of a tRNA molecule and that a specific nucleotide triplet elsewhere in the tRNA interacts with a particular triplet codon in mRNA through hydrogen bonding of complementary bases. A striking feature of the genetic code is that an amino acid may be specified by more than one codon, so the code is described as degenerate. This does not suggest that the code is flawed: although an amino acid may have two or more codons, each codon specifies only one amino acid. The degeneracy of the code is not uniform. Wobble allows some tRNAs to recognize more than one codon. Transfer RNAs base-pair with mRNA codons at a three-base sequence on the tRNA called the anticodon. The first base of the codon in mRNA (read in the 5’→3’ direction) pairs with the third base of the anticodon.

Crick proposed a set of four relationships called the *wobble hypothesis* [3,4,21]:

(1) The first two bases of an mRNA codon always form strong Watson-Crick base pairs with the corresponding bases of the tRNA anticodon and confer most of the coding specificity. The third position in each codon is much less specific than the first and second and is said to wobble.
(2) The first base of the anticodon (reading in the 5’→3’ direction; this pairs with the third base of the codon) determines the number of codons recognized by the tRNA. When the first base of the anticodon is C, base pairing is specific and only one codon is recognized by that tRNA. When the first base is U or G, binding is less specific and two different codons may be read. Adenine is very rarely used in this position and pairs mainly with U, but also with C and G. When inosine (I) is the first (wobble) nucleotide of an anticodon, three different codons (U, C, A) can be recognized.
(3) When an amino acid is specified by several different codons, the codons that differ in either of the first two bases require different tRNAs.
(4) A minimum of 32 tRNAs is required to translate all 61 codons (31 to encode the amino acids and one for initiation). Nowadays it is known that the smallest possible number of tRNA species with different anticodons able to read the genetic code of 20 amino acids is 26 [22]. However, 23 tRNAs are found in *S. cerevisae* mitochondria and 22 in certain organelles of vertebrates [22]. The wobble (or third) base of the codon contributes to specificity, but, because it pairs only loosely with its corresponding base in the anticodon, it permits rapid dissociation of the tRNA from its codon during protein synthesis. If all three bases of a codon engaged in strong Watson-Crick pairing with the three bases of the anticodon, tRNAs would dissociate too slowly and this would severely limit the rate of protein synthesis. It is now well established that there is a simple, linear trade-off between efficiency of cognate codon reading and accuracy of tRNA selection [23]. The maximal accuracy is highest for the second codon position and lowest for the third [23]. The third position in each codon is much less specific than the first and second and is said to wobble.

Further relevant biological properties of the tRNA code are:

(5) tRNAs are grouped into families of isoacceptors, with each family recognized by a single cognate aminoacyl-tRNA synthetase.
(6) All tRNAs conform to a secondary structure described as a “cloverleaf”, and fold in three-dimensional space into an “l-shaped” molecule, in which the amino acid and the anticodon are at opposite ends of the molecule.
(7) The 3’ end of all tRNAs have the sequence CCA, with the amino acid attached by the tRNA synthetase to the terminal adenosine residue. In eukaryotic cells, the 3’ terminal CCA is not encoded but is enzymatically added post-transcriptionally.
(8) During protein synthesis, tRNAs interact with the ribosomal “A” (aminoacyl), “P” (peptidyl) and “E” (exit) sites.
(9) All organisms exhibit preferred “codon bias”, in which certain synonymous codons are preferred over others, generally corresponding to cognate tRNA abundance.
(10) The human genome has 497 identified tRNA genes and 324 putative tRNA pseudogenes. There are no tRNAs that decode stop codons [16].
(11) An individual aminoacyl-tRNA synthetase must be specific not only for a single amino acid but for certain tRNAs as well.
(12) The coding rules appear to be more complex than those in the “first” code [8]. The genetic code describes translational assignments between codons and amino acids. tRNAs and aaRSs are those molecules by means of which these assignments are established.

Next, we will see that in the case of human tRNA set the assignments codon-anticodon [18] are remarkably different from the standard case [16,19].

## 5. Standard Hotel of tRNA Anticodons

### Some Previous Definitions

***Biologically complementary nucleotides***: We say that two nucleotides are biologically complementary if they share the same hydrogen bonds. So, C with G and U with A are biologically complementary. We observe that biological complementarity in the RNA code corresponds to those nucleotides that are connected in the double helix structure of the DNA code, that is, C with G and T with A.

***Reverse of a triplet***: We call the reverse of the triplet X_1_X_2_X_3_ the triplet X_3_X_2_X_1_, which is obtained one from the other by the interchange of the first nucleotide with the third one.

***The reverse complementary of a triplet or codon***: The reverse complementary of a triplet X_1_X_2_X_3_ is defined as the reverse of the triplet of its complementary nucleotides, that is, the triplet X_3_X_2_X_1_ where X_i_ denotes the complementary nucleotide of X_i_.

***The concept of anticodon***: The anticodon of a codon or triplet of tRNA is another triplet that recognizes it in the process of proteins synthesis. In most of the cases, the anticodon coincides with the reverse complementary of the codon.

***Function that converts each codon or triplet into its reverse complementary***: Consider the linear automorphism of the GF(4)-vector space NNN, whose matrix, with respect to the canonical basis is the orthogonal matrix P_13_=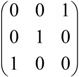 which leaves invariant the vector CUC and interchanges the vectors UCC and CCU. It represents the reflexion through the plane of equation x = z. Representing the triplet V = (X,Y,Z) as the column matrix V=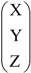 then, the matrix P_13_ converts V, under multiplication, into P_13_V=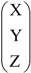, which is the column matrix associated with the reverse triplet (Z, Y, X). It is easy to note that the translation T_(3,3,3)_ associated with the vector GGG converts each triplet (X, Y, Z) into its complementary (X,Y,Z). Then the composed transformation T_(3,3,3)_◦P_13_ which is an isometric affine transformation, converts each triplet or codon into its reverse complementary or anticodon. Here we identify the matrix P_13_ with the linear automorphism it defines.

## 6. Codon-Anticodon Assignments of the Standard tRNA Code

**Figure 2:**
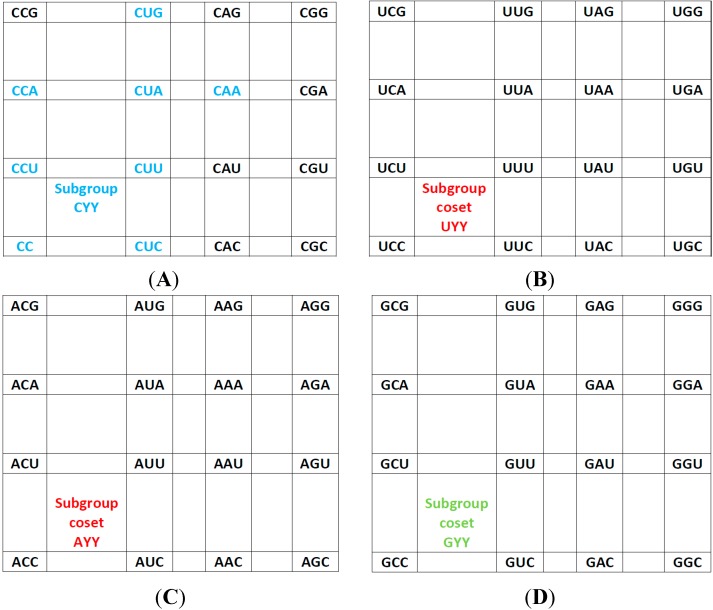
The subgroup cosets of the Standard Genetic Hotel. (**A**) The rear wall: set of triplets of type CNN. The subgroup with a tail, set SGwT, in the rear wall CNN; (**B**) The second inner wall: set of triplets of type UNN. It is a coset of the subgroup CNN, namely, UNN = CNN + UCC; (**C**) The first inner wall: set of triplets of type ANN. It is a coset of the subgroup CNN, namely, ANN = CNN + ACC (This is the set of triplets that are not anticodons of any codon in the S-tRNA-C); (**D**) The front wall: Set of triplets of type GNN. It is a coset of the subgroup CNN, namely, GNN = CNN + GCC.

Herein we describe the correspondences between codons and their anticodons in the standard tRNA code (adapted from [16]). In the following assignments, the arrows show, for each codon triplet at the left, its anticodon at the right. Both codons and anticodons are read in 5’ to 3’ orientation. It is shown that the sets of anticodons may differ from the expected (canonic) one-to-one correspondence. According to the SRM [16], attributions are successively fixed to tRNAs paired through the perfect palindromic anticodons, with the same bases at the extremities (5’ANA : UNU3’; GNG : CNC; principal dinucleotides underlined, pDiN). The 5’ degeneracy is then developed. The zeros in parenthesis mean that there are not genes for these anticodons,

***The rear wall:*** Set of codon triplets CNN that begin with C. See Figure 2A.

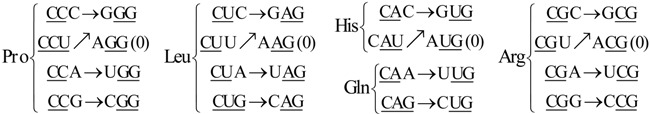



Here we see that there are four triplets: AGG, AAG, AUG, and ACG that are not utilized as anticodons (due to gene deletion), all beginning with A.

***The second inner wall:*** Set of codon triplets UNN that begin with U. See Figure 2B.

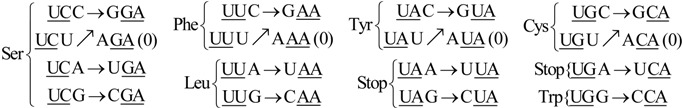



Observe that there are four triplets: AGA, AAA, AUA, and ACA that are not utilized as anticodons of any codon, all beginning in A.

***The first inner wall:*** Set of codon triplets ANN that begin with A. See Figure 2C.

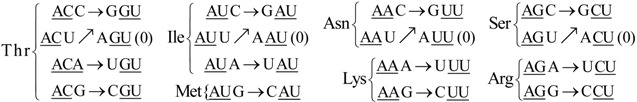



Note the absence of the four triplets: AGU, AAU, AUU, and ACU that are not utilized as anticodons of any codon, all beginning with A.

***The front wall***: Set of codon triplets GNN that begin with G. See Figure 2D.

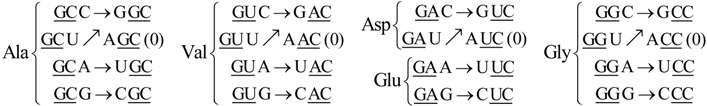



We remark that there are four triplets: AGC, AAC, AUC and ACC that are not utilized as anticodons of any codon, all beginning with A.

### 6.1. A General Conclusion about the Latter Assignments

We observe that, in the standard tRNA code that is reduced with respect to the expected full set of tRNAs, the 16 triplets that begin with A, that is, those of the first inner wall, are not utilized as anticodons of any codon (Figure 2C). Hence, all the triplets that begin with G, that is, those of the front wall, are shared anticodons, each by two triplets, one of them being its reverse complementary. The set of the standard tRNA anticodons is the set theoretical union *CNN*∪*UNN*∪*GNN*, of the rear wall CNN with the second inner wall UNN and the front wall GNN, that is, the union of the subgroup CNN of the additive group (NNN, +) with its two cosets UNN and GNN. The set of triplets that are not anticodons of any codon is the complementary set ANN, that is, the first inner wall, which is the remaining coset of the subgroup CNN.

### 6.2. The Three-Walls Hotel of the Standard tRNA Anticodons

**Figure 3:**
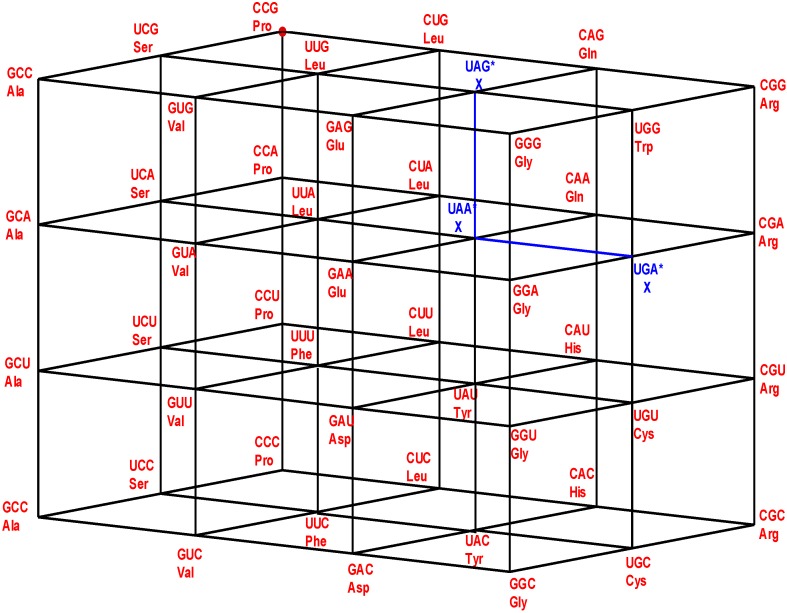
The Hotel of Anticodons of the S-tRNA-C (See text).

If, in the original Genetic Hotel NNN, the first inner wall, ANN, confirmed by the triplets that are not anticodons, is removed, we obtain a Hotel with three walls, confirmed by the sets CNN, UNN, and GNN. We will call it the Hotel of the Anticodons of the Standard tRNA code. The wall GNN is the set of shared anticodons (see Figure 3).

### 6.3. The Group of All the Symmetries of the Three-Walls Hotel of Standard tRNA Anticodons

We see that among the five generators A, B, T_GCC_, T_CGC_, T_CCG_ of the group E(NNN) of all the symmetries of the genetic hotel NNN, only B, T_CGC_, and T_CCG_ leave the three walls hotel *CNN*∪*UNN*∪*GNN* of standard tRNA anticodons invariant. Hence, they generate the subgroup of all the symmetries of it. It is an order eight group, with the defining relations:

B^2^ = (T_CGC_)^2^ = (T_CCG_)^2^ = I_3_,…, B◦ T_CGC_ = T_CCG_◦B, B◦ T_CCG_ = T_CGC_◦ B.

This group is isomorphic to the so-called quaternion group of eight elements.

## 7. Codon-Anticodon Assignments in the Human tRNA Code

Transfer ribonucleic acid (tRNA) decodes the genetic code by charging amino acids to the growing protein chain on the ribosome. With the availability of the complete sequence of the human genome at least 497 tRNA genes have been identified (which include some gene duplications) [18]. With the exception of the UGA-decoding tRNA that inserts SelCys into protein under special circumstances, no tRNAs were found that decode stop codons [18]. This opens up the possibility of using nonsense codons for insertion of nonnatural amino acids into selected proteins, to facilitate structural and functional analyses of these proteins. This has been accomplished by evolving tRNA:aminoacyl-tRNA synthetase pairs in which the synthetase recognizes a nonnatural amino acid and attaches it to its partner nonsense-suppressor tRNA and no other tRNAs. It has been noticed that in the human tRNA set [18], there are 16 triplets whose anticodons are not their reverse complementary, but the reverse complementary of a neighbor triplet, which differs from it only in the third nucleotide. The latter is shown next in the codon-anticodon assignments of the human tRNA (Section 7.1). We want to remark that these correspondences were adapted from [18]. This adaptation was necessary for a better understanding of our selected ordering {C,U,A,G} of the four RNA nucleotides.

### 7.1. Codon-Anticodon Correspondences in Human tRNA

Next, we enlist the assignments between codons and their anticodons in the human tRNA code. As before, in the following correspondences the arrows show for each triplet on the left, the according anticodon on the right. This shows, again, that the sets of anticodons may differ from the expected (canonic) one-to-one correspondence.

***The rear wall:*** Set of codon triplets CNN that begin with C. See Figure 2A.

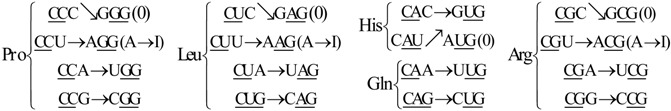



In this case we observe that there are four triplets: GGG, GAG, AUG, and GCG that are not utilized as anticodons (that is, there are no genes for them, marked zero), one of them beginning with A, and the other three beginning with G. Note that we have the same set of amino acids as those found in the S-tRNA-C but with different wobbling properties. Note also that the remaining 5’R anticodons have the transcribed As modified post-transcriptionally to Hypoxanthine (marked I, for the nucleoside Inosine). The pDiN in the triplets are underlined in order to facilitate visualization of the pairings. The notation here also applies to the following segments of the assignments.

***The second inner wall:*** Set of codon triplets UNN that begin with U. It is a coset of the subgroup CNN, namely, UNN = CNN + UCC. See Figure 2B.

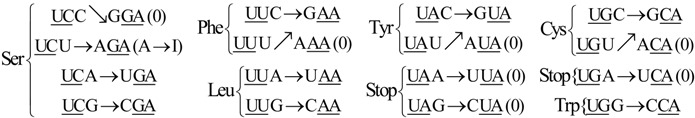



Note that there are four triplets GGA, AAA, AUA, and ACA, as well as the three stop triplets UUA, CUA, and UCA that do not exist, that are not utilized as anticodons of any codon. Again, this set of anticodons is not the same as those absent in the S-tRNA-C. In some circumstances, the codon UGA can be assigned to SelCys.

***The first inner wall:*** Set of codon triplets ANN that begin with A. See Figure 2C.

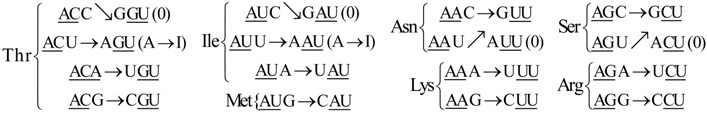



Observe that there are four triplets: GGU, GAU, AUU, and ACU that are not utilized as anticodons of any codon, two beginning with G and two beginning with A.

**Figure 4:**
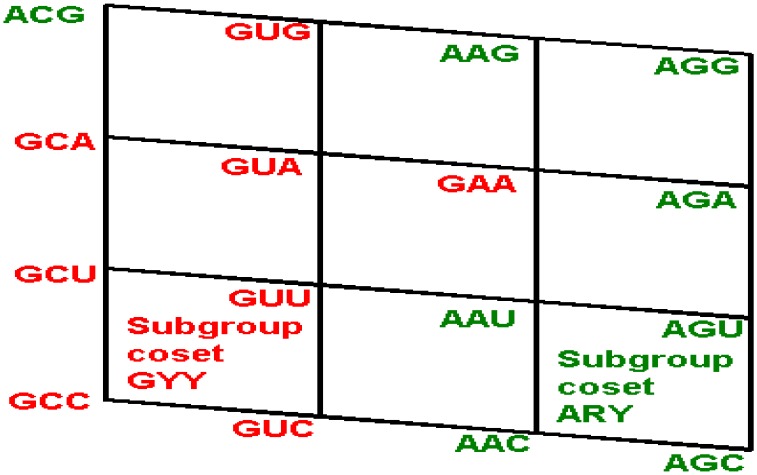
The shared front wall: Sets of triplets of type GNN (red) and of type ANN (green) that are shared anticodons in the human tRNA. The subgroup coset with tail in the front red wall GNN is the image under the isometric translation T_GCC_ of the subgroup with tail of the rear wall CNN. The subgroup coset with tail in the front green wall, ANN, is the image under the isometric translation T_AAU_ of the subgroup with tail of the rear wall CNN.

***The front wall:*** Set of codon triplets GNN that begin with G. See Figure 4.

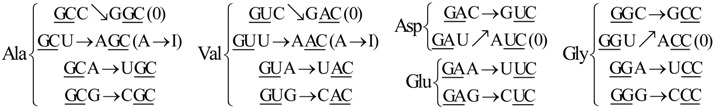



Note the absence of the four triplets: GGC, GAC, AUC, and ACC that are not utilized as anticodons of any codon, two beginning with G and the other two beginning with A.

### 7.2. A General Conclusion about the Latter Assignments

We observe that in the human tRNA set, compared to the standard set, there are also 16 triplets that are not anticodons of any codon, eight of them beginning with A, and the other eight beginning with G. Hence, there are 16 triplets that are shared anticodons, eight of which beginning with A, and the other eight beginning with G, each one being the anticodon of two triplets, one of them being its reverse complementary. The eight triplets beginning with G that are not anticodons in the human tRNA anticode are the images under the translation T_UCC_ of another eight triplets beginning with A that are not anticodons in the standard tRNA anticode. This set is the complement in the first inner wall, ANN, of the set of the eight triplets that are not anticodons in the human tRNA. Both sets are next to each other, with a common part of eight triplets that begin with A and two subsets of 8 eight triplets that begin with A, as is the case for the S-tRNA-C, and another eight triplets that begin with G in human tRNA anticode, which are pair-wise adjacent to those that begin with A.

Physiological considerations are necessary for understanding the diversity of mechanisms in decoding when utilizing the 5’R anticodons, which is not yet complete. The rationale for the 5’A elimination might encompass combinations of mechanisms. (1) The 5’R anticodons are of one kind only in each box, genes for the other kind are deleted (the zeroes). In the human genome, there are three cases with the exceptional presence of one tRNA gene each for the Ile GAU, Tyr AUA, and Asn AUU, which are considered possible pseudogenes. (2) In the standard tRNA code, it is considered that all genes for anticodons with 5’A would be deleted. While mechanisms for this *en bloc* deletion are not known, it could be supposed that tRNAs with 5’A anticodons would not be used due to some modification in the translation mechanisms or components that do not accept the 5’A anticodon-bearing tRNAs; therefore, the corresponding genes would be left free for pseudogenization or deletion. What is known so far is that the wobbling abilities of 5’A are harmful for the decoding of complex boxes (that is, with more than one attribution) since they can accept codonic C, U or G, while the activity of 5’G is of adequate specificity. In the human tRNA code the 5’A elimination was accomplished through this mechanism of gene deletion with specific retention of genes for 5’G triplets in eight cases: the seven dicodonic attributions of Phe, Cys, Ser, Tyr, His, Asp, Asn, plus the tetracodonic Gly the choice of which is difficult to explain. (3) In the other eight boxes of the human tRNA code the 5’A anticodons have the A’s modified to Hypoxanthine (nucleoside Inosine). The boxes overlap quite well with the set of simple boxes (with only one attribution), except for Gly, and adding Ile. It might be meaningful to physiology that the wobbling abilities of I are as wide as those of A, but modified to accept codons ending in C, U or A. The decoding of the Y bases remains with double possibilities and the 3’A codons now have these double possibilities, which might be adequate for low GC genes. The occurrence of the only case of a complex box receiving the A→I modification in the Ile box might suggest involvement of the initiation process on this choice, but this leaves the retention of the 5’G specificity in the Gly box unexplained.

#### 7.2.1. The First Main Conclusion

**Figure 5:**
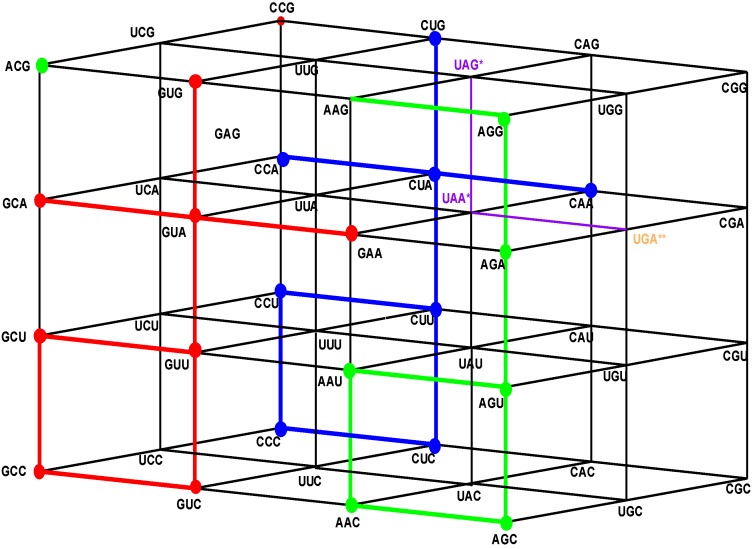
The Hotel of Anticodons in the H-tRNA-C. This is a hotel with only three front walls. The front wall is composed by GNN triplets. The 16 triplets in red and in green conform the wall of shared anticodons, joined in a unique set. The eight in red are those that begin with G and the eight in green are those that begin with A. The most external wall, anticodons of the type ANN, does not exist: it is the set of eight triplets beginning with A and eight that begin with G that are not anticodons of any codon.

Codons or triplets that share a common anticodon belong to the two first rows of each block, that is, they are triplets that end in a pyrimidine, C or U. Hence, they are situated on the first and the second floors of our Hotel of triplets, in a vertical segment. Additionally, we observe that triplets that share a common anticodon both specify the same amino acid. The set of codons that share their anticodons with neighbors is the subgroup NNY, the union of the first and the second floor NNC and NNU (see Figure 4 and Figure 5).

#### 7.2.2. The Second Main Conclusion

We see that the anticodons of codons that belong to the additive subgroup NNY, the union of the first and the second floors, all belong to the set RNN, the union of the front and the first inner wall GNN and ANN, which is a coset of the additive subgroup YNN, the image of NNY under the reflection P_13_. As the set RNY is the intersection of NNY with RNN, it is invariant under the action of the affine function *T*_(3,3,3)_·*P*_13_ of reverse complementarity, and also, under the non-bijective function that assigns the anticodons to every corresponding codon in the human tRNA anticode. The 16 triplets that are shared anticodons all belong to the set RNN, eight of them to the front wall GNN and the other eight to the first inner wall ANN. Namely, they are: GCC, GUC, GCU, GUU, GCA, GUA, GAA, GUG in GNN, and AAC, AGC, AAU, AGU, AGA, ACG, AAG, AGG in ANN. Notice that there are no adjacent elements between both sets.

### 7.3. A Remarkable Subset of the Rear Wall CNN

In order to develop the algebraic model of the H-tRNA-C we need now to consider the subset: SGwT = {CCC, CUC, CCU, CUU, CCA, CUA, CAA, CUG} of the rear wall CNN. The first four elements CCC, CUC, CCU, CUU, the rear square face of the cubic condominium YYY, conform to the additive subgroup CYY of NNN, and is isomorphic to the Klein Four Group. Three out of the other four elements of the set SGwT, namely CCA, CUA, and CUG belong to the coset CYR, obtained from CYY by the translation T_CCA_. However, the other subgroup, CAA, does not belong to the coset CYR, but to the coset CRR, another elementary square. For this reason, we will call the set SGwT a *Sub-group with a Tail*, and it explains the used notation. In Figure 5, we can see that the tail in blue, namely the subset {CCA, CUA, CAA, CUG}, that is not a subgroup coset, has the shape of a cross. The following result emerges:

***Theorem*:** The subsets of anticodons {GCC, GUC, GCU, GUU, GCA, GUA, GAA, GUA} of the front wall GNN, and {AAC, AGC, AAU, AGU, AGA, ACG, AAG, AGG} of the first inner wall ANN (Figure 4 and Figure 5), are the images of the sub-group with tail SGwT = {CCC, CUC, CCU, CUU, CCA, CUA, CAA, CUG} under the translations T_GCC_ and T_AAU_, respectively.

**Proof:** It follows, first, from the addition of the triplet GCC to each element of the set SGwT, and, second, from the addition to each element of the same set the triplet AAU. In fact:

{CCC, CUC, CCU, CUU, CCA, CUA, CAA, CUG} + GCC = {GCC, GUC, GCU, GUU, GCA, GUA, GAA, GUG}

while

{CCC, CUC, CCU, CUU, CCA, CUA, CAA, CUG} + AAU = {AAU, AGU, AAC, AGC, AAG, AGG, ACG, AGA} = {AAC, AGC, AAU, AGU, AGA, ACG, AAG, AGG}

reordering the set, he assertion is proved.          ■

An important observation: The translation T_GCC_ is isometric. For this reason, the image in red {GCA, GUA, GAA, GUG} of the tail in blue, {CCA, CUA, CAA, CUG}, (Figure 5), still has the shape of a cross. On the other hand, as the translation T_AAU_ is not isometric, the image in green, {AAG, AGG, ACG, AGA} of the tail, no longer has the shape of a cross (see Figure 4 and Figure 5).

A consequent terminology: As a natural consequence of the Theorem we will call here and further every subset {GCC, GUC, GCU, GUU, GCA, GUA, GAA, GUG}, and {AAC, AGC, AAU, AGU, AGA, ACG, AAG, AGG} of shared anticodons a *Subgroup Coset with a Tail.*

In the next sections we derive the corresponding Phenotypic Networks of amino acids for the SGC, S-tRNA-C, and the H-tRNA-C.

## 8. Phenotypic Graphs of Amino Acids

### 8.1. Equivalence Relation

A non-empty set *A* and a binary relation ℜ over *A* is an equivalence relation if the following properties hold:

(1) Reflexivity: Every element of *A* is in relation with himself: For all *x* ∈ *A*, *x*ℜ*x*.
(2) Symmetry: If an element of *A* is in relation with another element, the latter element is in relation with the former: For all *x*,*y* ∈ *A*, *x*ℜ*y* ⇒ *y*ℜ*x*
(3) Transitivity: If an element of *A* is in relation with another element, and the latter is in relation with a third one, then the first one is in relation with the last one: For all *x*,*y*,*z* ∈ *A*, *x*ℜ*y* ˄ *y*ℜ*z* ⇒ *x*ℜ*z*

The equivalence relation ℜ defines *A* different subsets named equivalence class, defined in the following way: Given *a* ∈ *A*,[*a*] = {*b* ∈ *A*|*a*ℜ*b*} This set is called the equivalence class associated with *a*.

The set containing all the equivalence classes is the quotient set and is denoted by *A*/ℜ The quotient set *A*/ℜ is a partition of the set *A*. This means that the set *A* can be divided into subsets, each subset formed only by equivalent elements from *A*. Formally, for a partition of *A* into the subsets {*A_i_*|*i*∈*I*}, the following properties hold:

(1) *A_i_* ≠ ∅, ∀*i* ∈ *I*.
(2)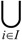 *A_i_* = *A*.
(3) *A_i_* ∩ *A_j_* ≠ ∅ ⇒ *A_i_* = *A_j_*.

### 8.2. The Only Twelve Ways of Representing the Phenotypic Graphs of Amino Acids

Given the set N = {C, U, A, G} of the nitrogenous bases, the set of all the triplets is the set NNN = {(X_1_X_2_X_3_)|X_1_, X_2_, X_3_ ∈ N}, and the set *A*∪{*S*} is defined, where *A* is the set of all the amino acids and *S* is the stop signal. It is natural to provide an equivalence relation ℜ to the set NNN defined in the following way: For (X_1_X_2_X_3_) (Y_1_Y_2_Y_3_) ∈ NNN, (X_1_X_2_X_3_) ℜ (Y_1_Y_2_Y_3_) ⇔ (X_1_X_2_X_3_) and (Y_1_Y_2_Y_3_) encode the same element in *A*∪{*S*} This relation, as seen before, induces a partition in the set where the equivalence classes are the subsets of codons that encode the same amino acid or stop signal, then a bijection can be defined between the set NNN/ℜ and the set of amino acids and the stop signal *A*∪{*S*}. This function is defined in the following way: *f*: NNN/ℜ → *A*∪{*S*} where [*a*]→the amino acid or stop signal that encodes *a*.

Example: The cube NNN can be seen as a graph *G* in which the set of vertexes or nodes *V*(*G*) represents the codons, and the edges or links will be assigned if two codons differ from one in only one of its entries.

#### 8.2.1. Graphs and Subgraphs

An undirected graph *G* is defined by a pair of sets *G* = (*V*, *E*), where *V* is a nonvoid countable set of elements, called vertices or nodes, and *E* is a set of unordered pairs of different vertices or nodes, called edges or links. The edge (*i*, *j*) joins the vertices *i* and *j*, which are said to be adjacent or connected. Connected vertexes are commonly called neighbors or nearest neighbors. The total number of vertices in the graph (the cardinality of *V*) is denoted as *N* and defines the order of the graph. For a graph of size *N*, the maximum number of edges is 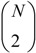 A graph with *E* = 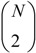, *i.e.*, in which all possible pairs of vertices are joined by edges, is called a complete *N*-graph. Undirected graphs are depicted graphically as a set of dots, representing the vertices, joined by lines between pairs of vertices, representing the corresponding edges. In an undirected graph the presence of an edge between vertices *i* and *j* connects the vertices in both directions.

From a mathematical point of view, a graph can be defined by means of the adjacency matrix X = {*x_ij_*}. This is a *N* × *N* matrix defined such that

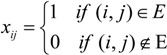



For undirected graphs the adjacency matrix is symmetric, *x_ij_* = *x_ji_*, and therefore contains redundant information.

#### 8.2.2. Subgraph

*A* set *G’* = (*V’*, *E’*) is said to be a subgraph of the graph *G* = (*V*, *E*), if all the vertexes in *V’* belong to *V* and all the edges in *E’* belong to *E*, *i.e.*, *E*' ⊂ *E* and *V*' ⊂ *V*.

The abundance of given types of subgraphs or cliques and their properties will be examined in Section 9.1, Section 9.2 and Section 9.3.

#### 8.2.3. Centrality Measures

In order to characterize the different graphs of the amino acids for the different codes we use the following statistical properties.

*Degree centrality*: The degree *k_i_* of a vertex *i* is defined as the number of edges in the graph incident on the vertex *i*.

For an undirected graph the degree measures how well an element is connected to other elements of the graph. For an undirected graph with a symmetric adjacency matrix, *k_in_*_,*i*_ = *k_out_*_,*i*_, where in-degree *k_in_*_,*i*_ of the node *i* is the number of edges arriving at *i*, while its out-degree *k*_*out*,*i*_ is the number of edges departing from *i*.

*Closeness centrality*: It expresses the average distance of a vertex to all others as

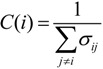



This measure gives a large centrality to nodes, which have the shortest path distances to the other nodes. This can be interpreted as how long it would take to spread information from a given node to all the other nodes sequentially.

*Betweenness centrality*: It is defined as the number of shortest paths between pairs of vertices that pass through a given vertex. More precisely, if *σ_hj_* is the total number of shortest paths from *h* to *j* and *σ_hj_*(*i*) is the number of these shortest paths that pass through the vertex *i*, the betweenness of *i* is defined as

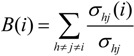



While the previous measures consider nodes which are topologically better connected to the rest of the network, they overlook vertices, which may be crucial for connecting different regions of the network by acting as bridges. The betweenness measures how many times a node is the path of least length across all the other nodes.

*Eigenvector:* This is the solution of the eigenvector problem *AV* = *λV*, where *A* is the adjacency matrix of the graph, *V* is the set of vertexes, and *λ* is the largest eigenvalue. This can be interpreted as the influence of a node in the graph, where a node will have a higher score if it is connected to high scoring nodes, and *vice versa*, even if the degree is equal.

## 9. Graphs of Amino Acids

### 9.1. Graphs of the SGC

As already pointed out in Section 2.2, there are 24 possible orderings of the four nucleotides that lead to 24 algebraic representations of the SGC either in 6D or in 3D [13]. Since each ordering of the nucleotides N = {C, U, A, G} yields a different cube, it is to be noted that given an ordering {X_1_, X_2_, X_3_, X_4_} of the nucleotides, the reverse ordering {X_4_, X_3_, X_2_, X_1_} leads to the same cube, differing from a reflection between the second and third floor and a rotation of 180° on the Z-axis. Therefore, the 24 algebraic representations of the SGC in 3D or 6D can be reduced to only 12 possible graphs for representing the codons of the SGC.

**Figure 6:**
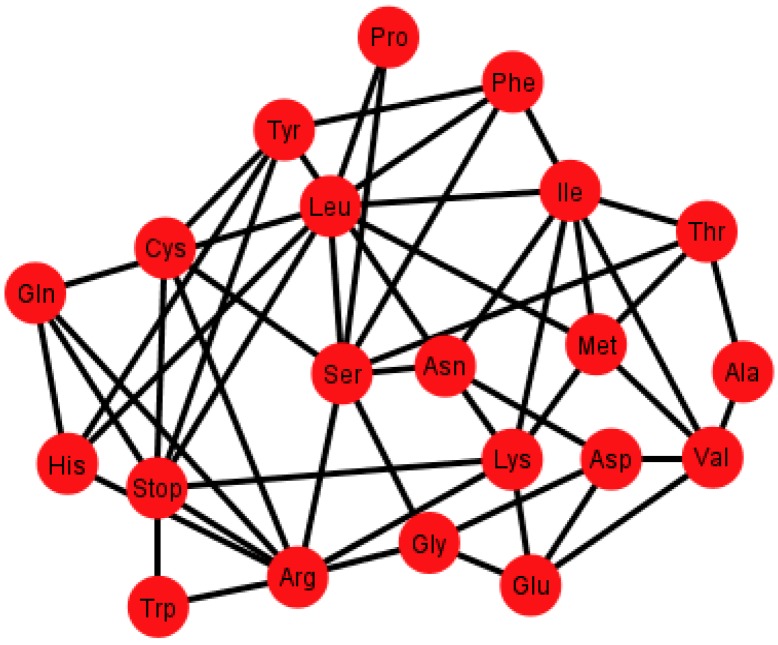
Graph *G_i_’* of the SGC corresponding to the ordering {C, U, A, G}.

The fact that the equivalence relation ℜ is a relation over the set *V*(*G*) implies that it is natural to build the quotient. This operation will yield a graph *G’* where the vertexes *V*(*G’*) will be the amino acids, and two amino acids will be neighbors if there exist two codons in the graph *G* that encode those amino acids that are neighbors. Since there are only 12 different representations of the graph *G*, each one of these representations will yield a different graph *G’*. The 12 different graphs of the SGC (*G’*), the S-tRNA-C (*T*), and the H-tRNA-C (*T’*), can be found in Supplementary Information SI-1, SI-2, and SI-3, respectively. As an example, Figure 6 shows the graph *G_i_’* corresponding to the ordering {C, U, A, G}.

Each graph *G’* has an associated adjacency matrix. For the SGC, it is worth to mention that the symmetrical adjacency matrix is not invariant for the different orderings. However, regardless of the ordering, all amino acids and the stop signal have at least one adjacency, *i.e.*, there is not an isolated vertex. For the SGC all graphs are connected, *i.e.*, all vertexes comprise a single communication class. Given any two amino acids there is always a path that connects them. A common fact on every graph *G_i_’*, *I* ∈ {1, 2, …, 12} (see SI-1) is that the edges: Gln–His, Phe–Leu, Ser–Arg, Stop–Tyr, Met–Ile, Lys–Asn, Glu–Asp are always present, *i.e.*, there is always an adjacency between their respective codons. The latter is due to the isometric groups previously described for every representation of NNN (see Section 2 and Section 3). In these groups there are codons of these amino acids that are neighbors on all representations of NNN. The 12 adjacency matrixes of *G’*, where each is a quotient associated with the SGC, can be found in SI-1. For the ordering {A, U, G, C}, for example, amongst the values of the degree of the different amino acids, Arg has the highest value of nine, which means that in this graph it is connected with nine other amino acids with a Hamming distance of one. In terms of codons, the codon for Arg is at nine non-synonymous mutations leading to nine different amino acids.

**Figure 7:**
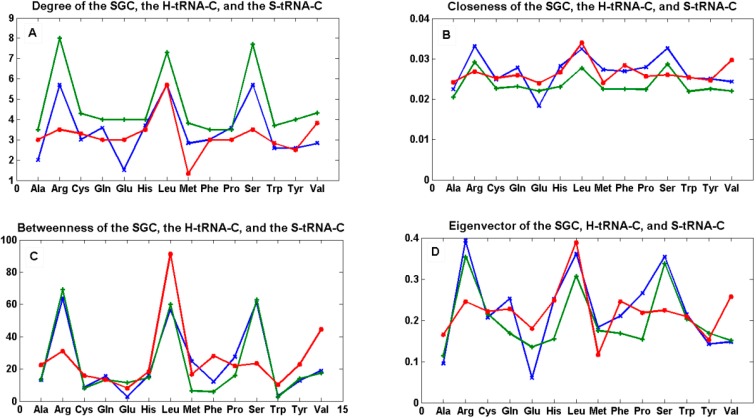
The mean of centrality measures. In (**A**) degree; (**B**) closeness; (**C**) betweenness; and (**D**) eigenvector of the mean of the 12 graphs *G_i_’* of the SGC (green), the mean of the 12 graphs *T_i_* of the S-tRNA-C (red), and the mean of the 12 graphs *T_i_’* of the H-tRNA-C (blue), *i* ∈ {1, …, 12}.

In order to compare the statistical properties of the 12 graphs for each code, we calculated the centrality measures for each of them and calculated their averages to perform a one-way ANOVA test to determine if they were statistically similar (Figure 7 and SI-4). We considered only the 14 amino acids, which were common to all three types of graphs in order to have a balanced ANOVA. This test was carried out using Matlab version R2012a, which uses the command “anova1”. If X is a matrix, anova1 treats each column as a separate group and determines whether the population means of the columns are equal. Note that the whisker plots for this test provide a test group of medians, and this is not to be confused with the F test for different means in the classical ANOVA table.

The centrality estimates of each of the 12 graphs for each code can be found in SI-1, SI-2, and SI-3.

We tested the null hypothesis that the patterns of all curves for each centrality measure were statistically indistinguishable. By means of a simple one-way ANOVA, we were unable to reject the hypothesis that the betweenness (*p* < 0.89) and eigenvector (*p* < 0.703) were statistically similar whereas we were able to reject the hypothesis that the degree (*p* < 0.012) and closeness (*p* < 0.022) were statistically similar. This simple finding means that the hubs (eigenvector) and the shortest paths (betweenness) are preserved among the three graphs, whereas the nodes are not all equally connected to the other nodes (degree), and the spread of information from a given node to all the other nodes (closeness) is not the same for all graphs.

We note that, in the case of the SGC, all its graphs *G_i_’* show that all the nodes have an almost uniform centrality. This means that the nodes have similar sums of distances to all the other nodes and, therefore, all nodes are uniformly spread in the graph.

### 9.2. Graphs of the S-tRNA-C

When considering only the standard tRNA code as a subset of the genetic code and constructing its graph, this will be a subgraph of *G*, and in every ordering of the nucleotides N, the quotient graphs of the tRNA *T_i_* will be a subgraph of *G_i_*. The betweenness across all the graphs of the standard tRNA *T_i_*, shows that the node Leu is usually the one with the highest betweenness, and Met and Trp are usually the ones with the lowest betweenness. The reason is that Leu is hexacodonic whereas Met and Trp are single coded.

A common factor of these graphs *T_i_* is that there exists a triangular prism on every graph except in two representations that correspond to the orderings: {C, U, A, G} and {G, A, C, U}, where only the bottom and top caps without walls exist, but are not connected (SI-2). The prism is a representation of the group ℤ_3_ × ℤ_2_ which is the product of two cyclic groups, and as a graph it is a six-vertex three-regular graph.

**Figure 8:**
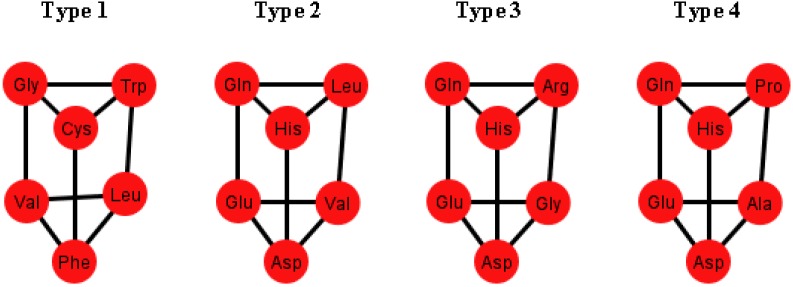
Diagrams of the four types of prisms that appear on the graphs *T_i_* that can be found in the S-tRNA-C.

These prisms come in four different types (Figure 8). As it can be seen, the last three types have the left wall as a common factor.

The distribution of the prisms across all the orderings is given in Table 2. Note, for example, that there are six orderings in which the prism type 1 can be found. The orderings {C, U, A, G} and {G, A, C, U} are skipped since these are the orderings that do not display a complete prism and only have the top and bottom caps. Almost all the amino acids are present on at least one type of prism but the amino acids Ser, Met and Tyr do not appear on any type of prisms. As it can be seen in Table 2, type 1 prism is the most common, but it is the one that does not have the common wall of the other types.

**Figure 9:**
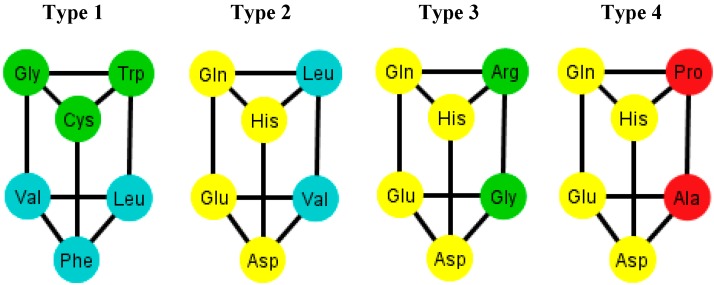
Diagrams of the four types of prisms that appear on the graphs *T_i_* where amino acids are colored according to their polar requirement values.

When considering the polar requirement scale values for each amino acid [24], these can be grouped into four colors. The colored prisms are represented in Figure 9, and the common walls of the prisms are in the same polar group (in yellow), and the other side of the prisms of the last three types range through the colors blue, green, and red.

**Table 2:** Distribution of the four types of prisms across the orderings of the nucleotides N.

	AUGC	AUCG	ACUG	AGUC	AGCU	ACGU	UAGC	UACG	UGAC	GUAC	Total
**Type 1**	**1**	**0**	**1**	**1**	**0**	**1**	**0**	**0**	**1**	**1**	**6**
**Type 2**	**1**	**1**	**0**	**0**	**0**	**0**	**1**	**1**	**0**	**0**	**4**
**Type 3**	**0**	**0**	**0**	**0**	**1**	**0**	**1**	**0**	**0**	**0**	**2**
**Type 4**	**0**	**0**	**0**	**0**	**0**	**1**	**0**	**1**	**0**	**0**	**2**

**Figure 10:**
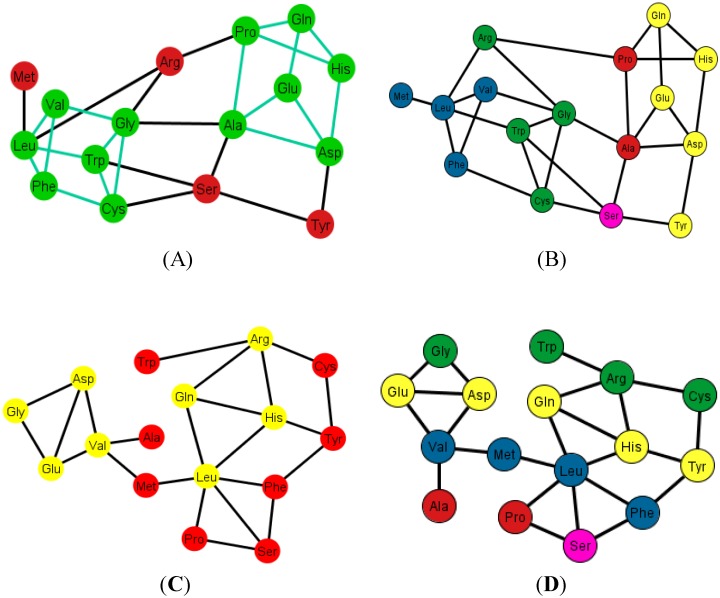
Graphs *T_i_* of the S-tRNA-C. In (**A**) and (**B**) the ordering is {A, C, G, U}. In (**A**) the prisms are highlighted in green; the prisms are of type 1 and type 4; in (**B**) the amino acids are colored according to their polar requirement values; In (**C**) and (**D**) the ordering is {C, U, A, G}. Note that the caps of the prisms are in yellow; the incomplete prisms are of type 2 and type 3; in (**D**) the amino acids are colored by their polar requirement scales. Ser is in pink in all graphs due to the fact that it has codons pertaining to two different polar requirement scales.

An interesting fact is that when the orderings of the graphs *T_i_* have all their prisms complete, they also have the amino acids separated accordingly by their polar requirement values (Figure 10). Note also that the orderings that have broken prisms or have two prisms joined by a wall, they do not separate the amino acids, but they can still be distinguished by their polar requirement scales.

### 9.3. Graphs of the H-tRNA-C

**Figure 11:**
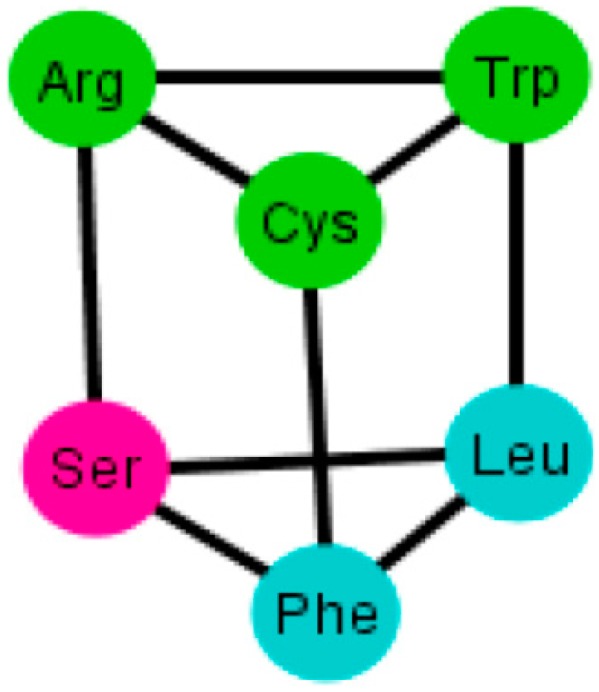
Diagram of type 5 prism present in only two arrangements of the human tRNA code.

For the case of the human tRNA code the same procedure is carried out such that there are 12 generated graphs, *T_i_’*, *i* ∈ {1, …, 12}, and in here only the arrangements {A, C, U, G} and {A, G, U, C} display a regular triangular prism. The prism present in these arrangements, from now on named type 5, is depicted in Figure 11. It is to be noted that it is almost equal to the type 1 prism but has Ser instead of Val. Recall that Ser is absent in the four types of prisms found in the graphs *T_i_* of the S-tRNA-C.

**Figure 12:**
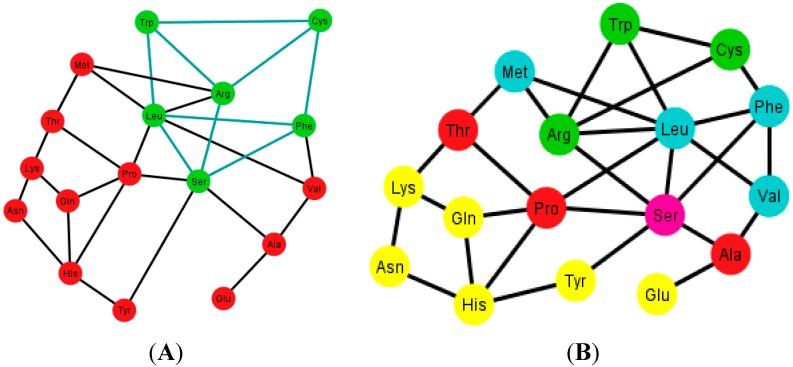
Diagrams of the human tRNA graph *T_i_’* according to the ordering {A, C, U, G}. In (**A**) the prism is colored in green; the prism is of type 5, different from the ones found in the graphs *T_i_*, of the S-tRNA-C; In (**B**) the amino acids are colored according to their polar requirements except for Ser that is in pink due to the fact that it has codons of different polar requirement scales.

As an example, consider Figure 12A that shows the graph associated with the arrangement {A, C, U, C}. Figure 12B shows the same graph but with the amino acids colored according to their polar requirements. Note that in the latter graph there are no neat disjoint clusters of amino acids. Only in the case of the ordering {A, G, U, C} is the graph disconnected by Glu.

When this procedure is applied to the SGC, the graphs *G_i_’*, *i* ∈ {1, …, 12} will have as subgraphs *T_i_*, *T_i_’* and so will also have the prisms corresponding to the type of ordering. In other words, the arrangement *i* will have all the prisms of *T_i_* and *T_i_’* with *i* ∈ {1, …, 12}.

## 10. Discussion

Novel 3D algebraic models of the S-tRNA-C and H-tRNA-C have been rigorously derived, and we compared them with the Genetic Hotel of the SGC. The symmetry groups of the three-walls Hotels of the SGC, the S-tRNA-C, and the H-tRNA-C have been determined. The most symmetric of these genetic codes is the SGC followed by the S-tRNA-C, and the least symmetric is the H-tRNA-C. In fact, we had to create a new algebraic concept, the subgroup with a tail, in order to describe the latter. The SGC can be broken down into a product of simpler groups reflecting the pattern of degeneracy observed [12,14]. Both the S-tRNA-C and the H-tRNA-C map onto the same SGC via different idiosyncrasies of their tRNAs by means of wobbling. It is worth mentioning that there are slight differences between the codon-anticodon assignments in the human tRNA code with those of other primates such as chimpanzee, rhesus macaque, and gorilla [25]. These different sets of tRNAs of the primate family are currently being examined in our group.

We also showed that there could be only 12 different graphs for representing the corresponding amino acids for each code. These 12 ways of representing the networks of amino acids depend on the ordering of the four RNA nucleotides. The averages of the centrality measures of the 12 graphs for each of the three codes were calculated. A simple one-way ANOVA test showed that the eigenvector and betweenness were statistically indistinguishable in the three types of graphs while the degree and closeness differed from each other. Therefore, while the topology of the three graphs is statistically the same, the differences in degree and closeness essentially capture the idiosyncrasies of wobbling of the S-tRNA-C and the H-tRNA-C in regard to the SGC.

The asymmetrical H-tRNA-C is also reflected by the fact that its phenotypic graphs display only two common prisms of amino acids in 10 out of the 12 possible graphs. In contrast, in the S-tRNA-C there are 10 out of the 12 possible graphs, which share a common type of prism. These subgraphs are relevant in the characterization of the networks of amino acids for the different genetic codes. For example, when the triangular prisms were colored according to the physicochemical property of polar requirement of amino acids [24], interestingly, the prisms showed uniform colors in some of their faces. The amino acids separated by their polar requirement scales coincide with the orderings that have all their complete prisms. We note that the polar requirement values of amino acids have been assigned to their respective codons in the classical table of the genetic code [24,26]. Therefore, to our knowledge, this is the first time that polar requirement values are directly assigned to amino acids in a network. It would stand to reason that Ser and Met are missing codons on the prisms of the graphs *T_i_*, because Met has only one codon, whilst Ser has codons on different scales of polar requirement, and therefore they would break the symmetry. However, we cannot use the same argument to explain the presence of Ser on the prism type 5 of the graphs *T_i_’*. The difference lies in the fact that Ser in the S-tRNA code has as anticodon GGA which recognizes the codons UCC and UCU whereas in the H-tRNA-C the anticodon AGA binds to both codons UCC and UCU that pertain to different polar requirement classes. We also used the hydropathy values of the amino acids [27], and it turned out that these values did no form clear color patterns that matched the triangular prisms. This may be due to the fact that Gly-CC: Pro-GG and Ser-GA:Ser-CU correspond to hydropathy outliers [27]. The most parsimonious explanation for the absence/presence of Ser-Met-Tyr is provided by the SRM [16,19]. Polar requirement is an abiotic property of free amino acid molecules in solution; hydropathy is a substitute for that term but refers to properties of amino acid residues in protein tertiary structures, such as belonging to hydrophilic or hydrophobic stretches or segments or regions of molecules that, due to this, will be internal or external in the globular proteins or transmembranal or extramembranal in membrane proteins. Therefore, hydropathy is a biologic property [27]. There are three groups in the hydropathy correlation: the first consists of non-correlated hydroapathetic amino acids; the second correlates are the extremes, the highly hydrophilic amino acids together with their partners that create dimers that are highly hydrophobic; the third belongs to the mixed sector of triplets and presents a nice line filled throughout its whole length. The dimer rationale presupposes the “phenotypic groups” to be hydropathy-discrepant, as we have found in this work.

**Figure 13:**
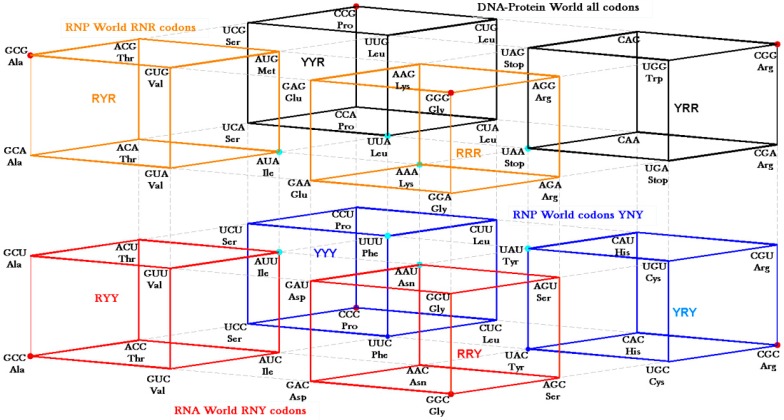
Genetic Hotel of the SGC and its evolution, See text.

The RNY codon model that was utilized for the algebraic procedure of the Hotels has biological backing in the observations of Eigen’s group on tRNA sequences [10] who supposed them to be ancient genes. The abiotic support comes from the observations on abundant amino acids in Miller’s sets [28,29] and in Trifonov’s review [30] that concentrate on the GNC row of the matrix. Symmetry procedures are added by the Hotels procedures to expand the initial RNY set to reach the full set of codes. In fact, the evolution of the SGC from a primeval RNY code can be easily visualized from the Genetic Hotel, as illustrated in Figure 13. The set of codons RNY comprises what is called the RNA World (red). At this stage, there were 16 RNY triplets encoding eight amino acids. The products were ribozymes and coenzymes that were used for obtaining energy (NADP, FAD, ATP-synthase). Most of these molecules were RNY sequences. Simple translations of this RNY condominium lead to the sets YNY (blue) and RNR (orange) that altogether formed the Ribo-Nucleoprotein World. The second tRNA code, currently known as the Second Operational tRNA code, appeared at this stage. This second code is an analog and non-degenerate code (20 amino acids charged by 20 aatRNAs). The duplication of the first half of proto-tRNA minihelixes gave rise to the third tRNA code of digital nature: codon-anticodon interactions. Finally, a translation of any of the condominiums YNY and/or RNR lead to the set of YNR codons (black) which altogether originated the DNA-Protein World (the four condominiums). The Last Universal Common Ancestor is considered to be a population of organisms possessing this frozen code [26,31]. It is worth to mention that the predictions of critical scale invariance (using renormalization group techniques) associated with symmetry breaking at the different stages of the evolution of the SGC have been verified with actual data of current genomes of Eubacteria and Archaea [15].

There are two ways of deriving the SGC from the primeval RNY code. First, we considered not a strict comma-less code as proposed by Crick *et al.* [32] but rather a degenerate RNA code which could be translated in the first (RNY), second (NYR), and the third (YRN) reading frames (frame-shift reading mistranslations that allow for the so-called statistical proteins [24]). The second pathway, shown in Figure 13, is derived by allowing transversions in the first (YNY) and third (RNR) nucleotide bases of the 16 codons of the RNA code. It is worth mentioning the discovery of the so-called circular codes [33], which have several remarkable properties ([34] and references therein). For example, it has recently been shown that circular codes show imprints in motifs of tRNAs and in 16S rRNA [35]. A maximal circular code *X_o_* of 20 trinucleotides was identified statistically on a large gene population of eukaryotes and prokaryotes [33]:

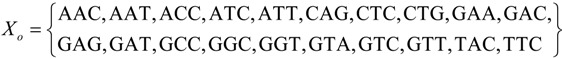



We remark that there is an interesting and intriguing connection between the circular codes with our approach of frame-shift reading mistranslations for obtaining the SGC from RNY codons. Note in fact that there are 12 out of the 16 RNY codons in *X_o_* and this circular code has been related to the origin of a primeval genetic code [36].

The SRM is only now starting to be examined with mathematical procedures. Danckwerts and Neubert [37] used the Four-Klein group to partition the set of dinucleotides into the doublets that would match with a third base for a triplet that has no influence on the coded amino acid (M1 set) and those doublets that do not code for amino acids without knowledge of the third base in the triplet (M2 set). This approach is directly applicable to the SRM, which is based on pDiN. In the SRM the set M1 = {GU, GG, AG, CG, GA, GC, AC, CC} read in 3’ to 5’ for anticodons clearly forms a Four Klein Group. In the SRM, the set {GU, GC, AC, CC} belongs to the mixed YR pDiN, whereas the set {GG, AG, CG, GA} corresponds to the homogeneous sector of RR pDiN, The SRM starts exactly on the basis of symmetries derived from triplet complementarity and is very simple, with encodings occurring in four modules of four boxes each, dictated neatly by ∆G-values. A chronology of amino acid encodings was generated on the basis of various protein functional properties. The chronology indicated a starting couple of amino acids, Gly and Ser, which was not suspected by any of the previous models but found support in a simple amino acid biosynthesis pathway. The suggestion received further consistency from independent phylometabolic examinations [38] but still waits for experimental tests on the dimer mechanism. 

There are many algebraic models for the formation of the code. A pioneering work of the use of group theory for extracting the symmetries and evolution of the SGC was developed by Hornos and Hornos in 1993 [39]. Their approach, which uses Lie algebras, is based on analogies with particle physics and symmetry breaking from higher-dimensional space [40]. They showed that the current SGC must be slightly broken and they successfully predicted experimental values of amino acids polarities. Another most recent model, which also uses Lie algebras of the genetic code over the Galois field of four DNA bases, has been developed [41]. With our model, based on elementary algebra, we have shown that with simple translations of the RNY condominium we can derive the whole SGC. We also highlight two more non-mathematical models that are apparently the reverse of each other. (a) A couple of synthetases in one tRNA. The observations made by Ribas de Pouplana and Schimmel [42] that specific pairs of aaRSs—one from each of their two classes—can be docked simultaneously onto the acceptor stem of tRNA point to interactions that may have existed between the aaRSs ancestors using a reduced set of tRNAs. (b) A couple of tRNAs with transferase activity. The SRM proposes that tRNAs would form dimers by pairing through the anticodons and that the dimers of aaRSs would be able to transfer the amino acids from one to the other, starting the protein synthesis process [16,19]. The Serine codes, that are enigmatic for all other models, would be representatives of an early situation where the two tRNAs in a dimer (anticodons wobble-G-A:wobble-C-U) would code for the same amino acid. Following a similar rationale, a conflict (due to a direct and/or reverse complementarity) or more complex relationships are suggested between the initiation and the Tyrosine codes (Met codon AUG *versus* Tyr codons UAY).

Since the evolution of the genetic code is apparently a problem of symmetry breaking, we contend that the H-tRNA-C is in an ongoing evolution whereas the S-tRNA-C is more in a frozen state. The latter preserves symmetry, for example, in the formation of the four isometric subgroup cosets CYY, UYY, AYY, and GYY (Figure 2) whereas in the former we have two subgroup cosets with a tail, one of them is the isometric GYY, whereas the other subgroup, ARY, is not isometric (Figure 4). In addition, in the dimer approach, the SRM [16], each module of triplets is a perfectly symmetric structure: module 1 is wGR:wCY, where w is one purine (G in the standard, G or I in the human) plus the two pyrimidines; module 2 is wAR:wUY; module 3 is wGY:wCR; module 4 is wAY:wUR.

The S-tRNA-C is present in organisms that are not intensely regulatory, like Archaeas. Humans are an example of organisms that are more intensely regulatory, such as eukaryotes or vertebrates.

The paramount importance of tRNA for the evolution of the genetic code cannot be understated. It has recently been shown that the origin and evolution of the Peptidyl Transferase Center (PTC) of the ribosome could have evolved from proto-tRNAs [43].

## 11. Conclusions

The symmetry groups of 3D algebraic models of the SGC, the S-tRNA-C, and the H-tRNA code have been determined. The evolution of the codes can be reflected by successive symmetry breakings. Both the SGC and the S-tRNA code are more symmetrical than the H-tRNA code which means that the former are in a frozen state whereas the latter is still evolving. We demonstrated that there are only 12 ways for representing the phenotypic networks of amino acids for each code. The physicochemical property of polar requirement overlaps with the general topology of the networks of amino acids. We show that the degree and closeness of these networks capture the idiosyncracies of wobbling of the S-tRNA-C and the H-tRNA-C. The evolution of the whole Genetic Hotel of the SGC can be neatly reconstructed by means of simple translations of the RNY primeval genetic code.

## Supplementary Files

Supplementary File 1
